# Image analysis data for the study of the reactivity of the phases in Nd-Fe-B magnets etched with HCl-saturated Cyphos IL 101

**DOI:** 10.1016/j.dib.2020.106203

**Published:** 2020-08-20

**Authors:** Martina Orefice, Xuan Xu, Kristina Žužek Rožman, Sašo Šturm, Koen Binnemans

**Affiliations:** a KU Leuven, Department of Chemistry, Celestijnenlaan 200F, P.O. Box 2404, B-3001 Heverlee (Belgium); b Jožef Stefan Institute (JSI), Department for Nanostructured Materials K7, Jamova Cesta 39, SI-1000 Ljubljana (Slovenia)

**Keywords:** Nd‒Fe‒B magnets, Corrosion, ImageJ, Image analysis, Scanning-electron microscopy, Etching, Ionic liquids, Rare earths

## Abstract

Three phases can be distinguished in Nd‒Fe‒B permanent magnets: a Nd_2_Fe_14_B matrix grain phase, a Nd-rich grain boundary phase and a Nd-oxide phases. Common reaction models for leaching, such as the shrinking-particle model, cannot simply be applied to composite Nd‒Fe‒B permanent magnets because of the different chemical reactivities of the crystalline phases mentioned above. Etching the surface of a Nd‒Fe‒B magnet to expose its microstructure to electron microscopy is a necessary practice to correlate the microstructure itself to the specific properties of the magnets. Aqueous solutions of mineral acids are often used for etching purposes. However, these solutions are too low viscous to easily control the etching front and they show little selectivity in the etching process. In our work, the ionic liquid Cyphos IL 101 was used to etch bulk magnets instead of aqueous HCl solutions. The bulk Nd‒Fe‒B magnets were first polished, then exposed to a solution of 3 M HCl in Cyphos IL 101 for different times and at different temperatures. Afterwards, the etched Nd‒Fe‒B magnets were washed with ethanol and acetone. The results were examined via scanning-electron microscopy and image analysis. A commercial software, ImageJ®, was employed for image analysis. The latter technique was used to correlate the etched area (%area) or the grain and oxide size to the etching temperature or the etching time. The grain or the oxide size were calculated as Feret diameter. Image analysis revealed to be a necessary tool to support and correct the findings first suggested by the simple scanning-electron microscopy. The data presented in this article might be reused to corroborate a new reactivity order of the three Nd‒Fe‒B phases, different from that traditionally reported in literature, which is – from the most to the least reactive – grain boundary > oxides > the Nd_2_Fe_14_B grain phase.

**Specifications Table**

**Table:** 

Subject	Metals and alloys
Specific subject area	Corrosion of Nd‒Fe‒B permanent magnets and image analysis
Type of data	Tables – Images – Graphs
How data were acquired	ICP-OES (Perkin Elmer Optima 8300); SEM and EDS (JEOL JSM 5800 microscope); image analysis (ImageJ® software)
Data format	Raw – Analyzed – Fitted data
Parameters for data collection	SEM–EDS: Microscope operating at 20 kV; images acquired in back-scattered mode; EDS calculated on the average of 5 points per SEM image; Image analysis: data analysed: Feret diameter and *%area*; images in greyscale: 8-bytes (0-255 grayscale);
Description of data collection	SEM–EDS: samples were made conductive by spraying a carbon layer on them using a Balzer SCD 050 sputter coater Image analysis: three SEM pictures analysed for each experimental condition; two operators performing the analysis (to minimize human error); threshold referred to the particles area for the calculation of the Feret diameter and to the void area for the calculation of the etched area (%area)
Data source location	Institution: Jožef Stefan Institute City: Ljubljana Country: Slovenia
Data accessibility	“With the article” The raw data for the image analysis are on repository Repository name: [Zenodo] Data identification number: [10.5281/zenodo.3753084] Direct URL to data: [doi.org/10.5281/zenodo.3753084]

**Value of the Data**

These data give a new insight in the reactivity of the three phases of Nd‒Fe‒B magnets, the stoichiometric grain phase, the oxide phase and the grain boundary phase.
Materials scientists and engineers involved in the production of Nd‒Fe‒B magnets can benefit of these data for the design of new magnets and/or for the study of Nd‒Fe‒B magnets corrosion.
These data can be integrated with similar ones from the corrosion of Nd‒Fe‒B magnets in other media, such as nitric acid, aqueous saline solutions, to give a general scheme for the reactivity of the three phases of Nd‒Fe‒B magnets.
A technique cheaper than those commonly used to reveal the Nd‒Fe‒B magnet surface has been proposed in this paper.

## 1 Data Description

**Fig. 1:**
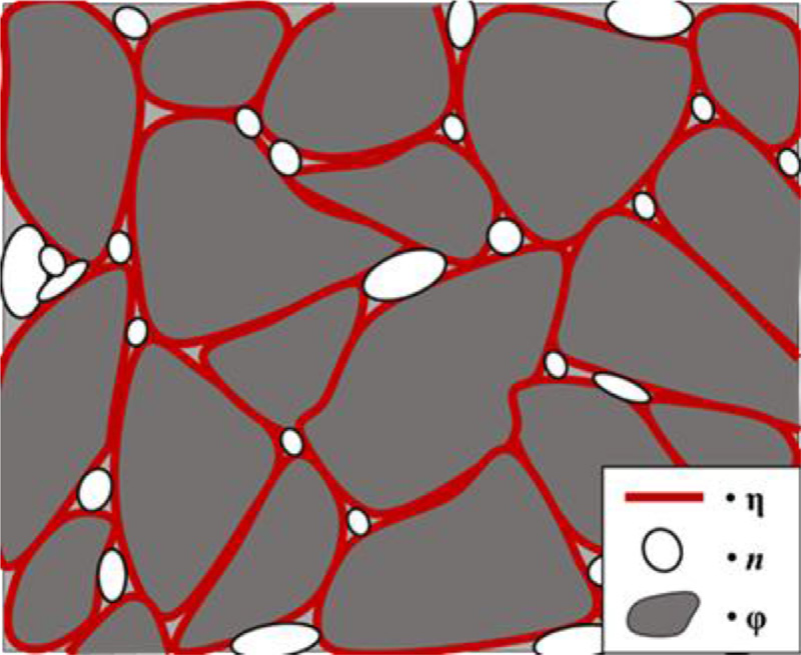
Composite microstructure of a Nd‒Fe‒B magnet. η = grain boundary, *n* = oxide grains and φ = Nd_2_Fe_14_B grain phase

**Fig. 2:**
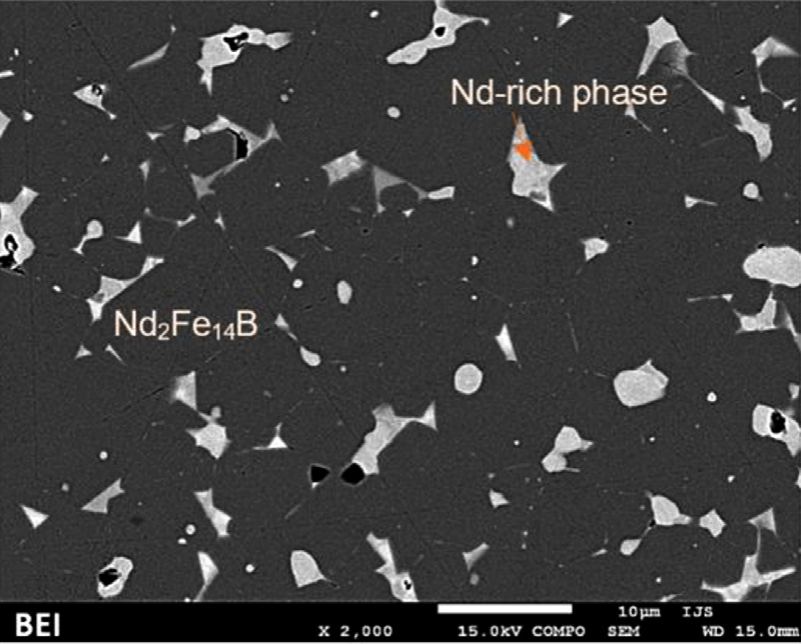
Back-scattered electron image of unetched Nd‒Fe‒B magnet.

A simple scheme of the microstructure of Nd‒Fe‒B magnets is shown in Fig. 1: the grain boundary η (red), the Nd-rich oxides *n* (white) and the stoichiometric Nd_2_Fe_14_B phase φ (grey). The elemental composition of the treated Nd‒Fe‒B magnets has been previously obtained [1]. Table 1 reports the composition per major elements of the phases φ (Fe, Nd and O – the latter being a contamination) and *n* (Nd, Dy and O), obtained via EDS; Fig. 2 is a back-scattered electron image of an unetched Nd‒Fe‒B magnet.

**Table 1:** Composition of the φ and *n* phases of the unetched sample, determined by EDS.

φ phase	at. %	st. dev. (%)	*n* phase	at. %	st. dev. (%)
O	4.2	6.1	O	23.3	27.5
Fe	86.9	0.5	Nd	61.9	8.4
Nd	9.0	3.2	Dy	14.9	10.7

**Fig. 3:**
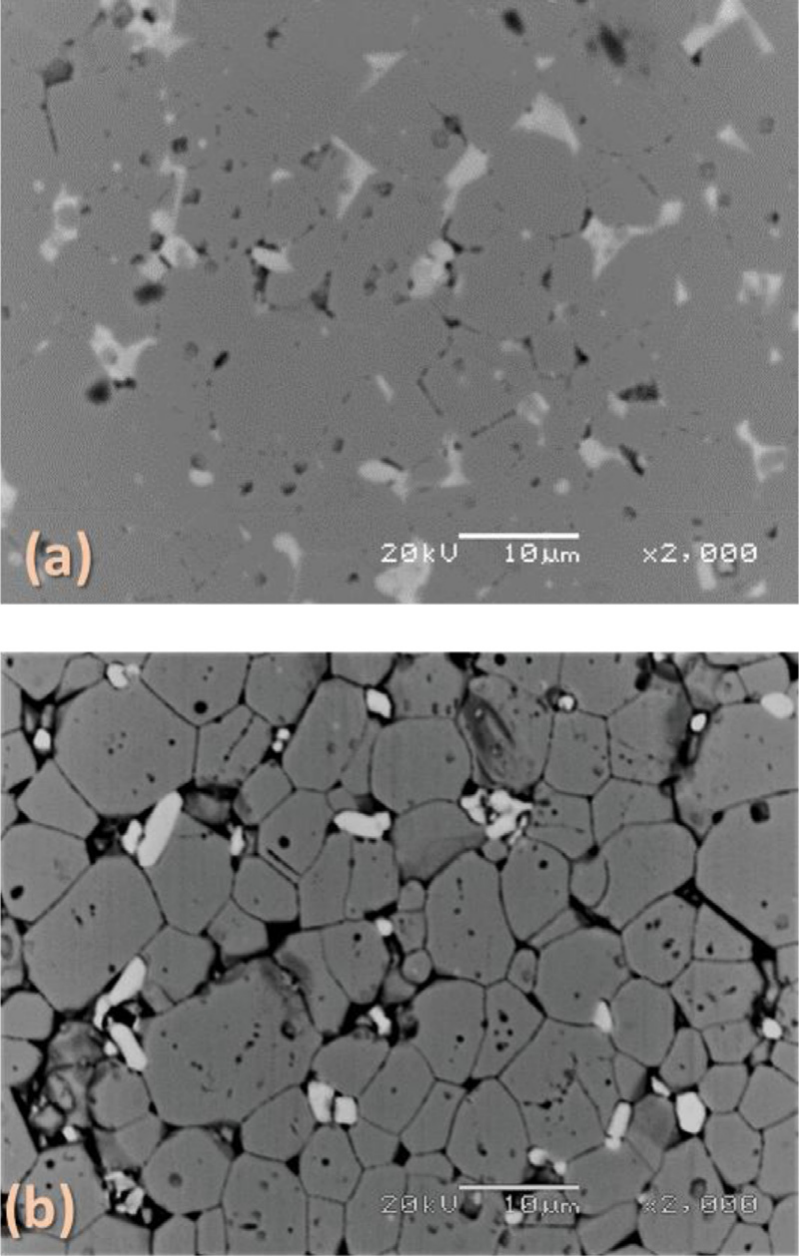
(a) and (b). Back-scattered electron image of a Nd‒Fe‒B magnet etched at room temperature for 10 min in: (a) 1.2 M HCl in Cyphos IL 101; (b) 3 M HCl in Cyphos IL 101.

**Fig. 4:**
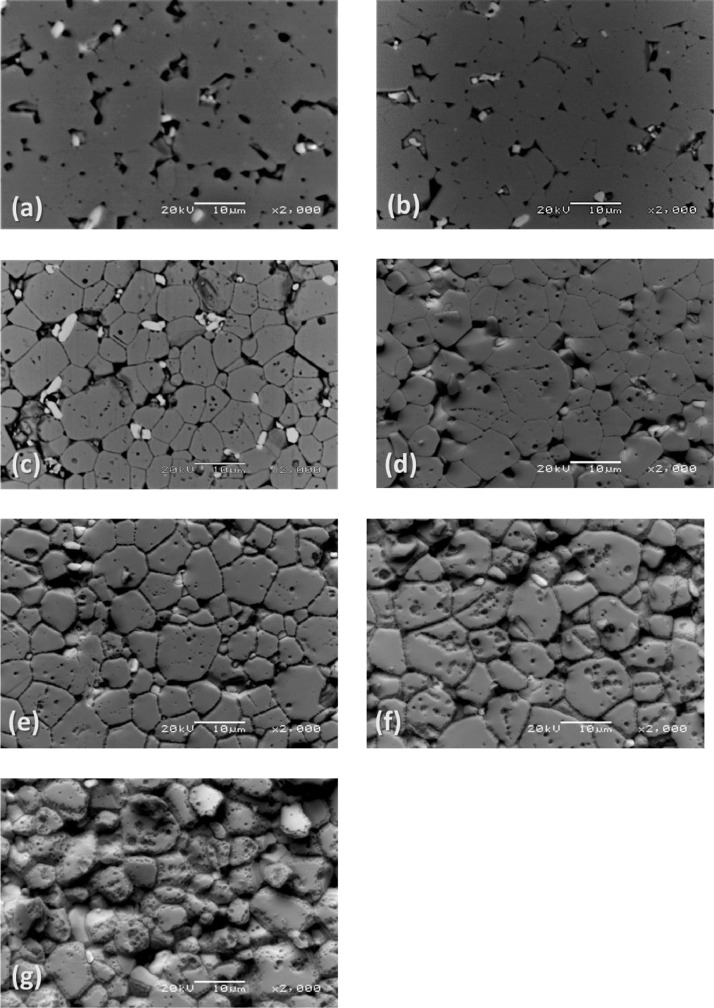
(a–g) Back-scattered electron image of a Nd‒Fe‒B magnet etched at room temperature and with 3 M HCl for: (a) 1.5 min, (b) 3 min, (c) 10 min, (d) 30 min, (e) 60 min, (f) 120 min and (g) 240 min.

**Fig. 5:**
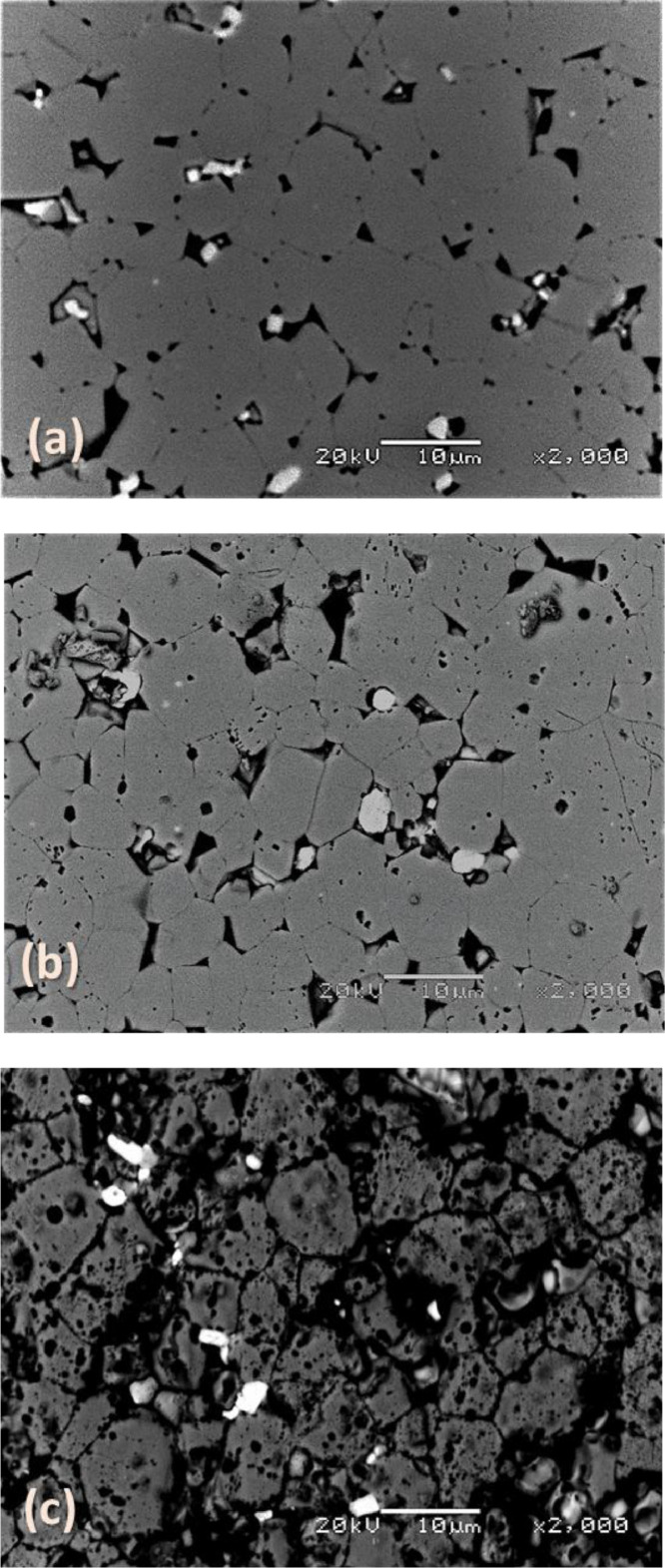
(a–c). Back-scattered electron image of Nd‒Fe‒B magnet etched for 3 min with 3 M HCl at: (a) room temperature, (b) 40°C, (c) 60°C.

Fig. 3, Fig. 4, Fig. 5 report back-scattered electron images of the effect of the HCl concentration in Cyphos IL 101; of the etching time and of the temperature on the etching of Nd‒Fe‒B magnets. In particular, Fig. 3 (a) and (b) depict the effect of the HCl concentration – (a) 1.2 M and (b) 3.0 M – in the Cyphos IL 101 on Nd‒Fe‒B magnets etched for 3 min at room temperature. Fig. 4 (a-g) show the effect of the etching time – (a) 1.5 min, (b) 3 min, (c) 10 min, (d) 30 min, (e) 60 min, (f) 120 min and (g) 240 min – on Nd‒Fe‒B magnets etched at room temperature with 3 M HCl in Cyphos IL 101. Tables 2 and 3 report the EDS composition of the φ and *n* phases, respectively, as a function of the etching time for samples etched at room temperature with 3 M HCl in Cyphos IL 101. In the latter Tables, the EDS composition of the φ and *n* phases of the samples etched for 120 min at 60°C with 3 M HCl in Cyphos IL 101 is reported for comparison.

**Table 2:** EDS composition of the φ phase as a function of the etching time after etching with 3 M HCl in Cyphos IL 101 at room temperature. The results of the sample etched for 120 min at 60°C are added for comparison.

t (min)		O	Fe	Nd
0*	at. (%)	4.2	86.9	9
	st. dev. (%)	6.1	0.5	3.2
1.5	at. (%)	4.5	86.7	8.8
	st. dev. (%)	6	0.7	6.4
3	at. (%)	4.3	86.9	8.8
	st. dev. (%)	18	0.6	2.5
10	at. (%)	4.9	86.4	8.7
	st. dev. (%)	23.2	1.3	0.9
120 (60°C)	at. (%)	6.6	84.5	9
	st. dev. (%)	14.4	1.3	1.8

⁎ sample 0 refers to the unetched sample.

**Table 3:** EDS composition of the *n* phase as a function of the etching time after etching with 3 M HCl in Cyphos IL 101 at room temperature. The results of the sample etched for 120 min at 60°C are added for comparison.

t (min)		O	Nd	Dy
0*	at. (%)	23.3	61.9	14.9
	st. dev. (%)	27.5	8.4	10.7
1.5	at. (%)	28.2	58.4	13.4
	st. dev. (%)	6.6	3	6.6
3	at. (%)	21.2	63.4	15.4
	st. dev. (%)	45.2	12.1	15.4
10	at. (%)	26.5	60.5	13
	st. dev. (%)	4.1	3.2	6.9
120 (60°C)	at. (%)	23.4	53.9	16.1
	st. dev. (%)	59.6	8.2	10

⁎ sample 0 refers to the unetched sample.

Fig. 5 (a-c) depict the effect of the temperature – (a) room temperature, (b) 40°C and (c) 60°C – on Nd‒Fe‒B magnets etched for 3 min with 3 M HCl in Cyphos IL 101. Tables 4 and 5 report the EDS composition of the φ and *n* phases, respectively, as an effect of the etching temperature for samples etched for 3 min with 3 M HCl in Cyphos IL 101. As for Tables 2 and 3, also in Tables 4 and 5, the EDS composition of the φ and *n* phases of the samples etched for 120 min at 60°C with 3 M HCl in Cyphos IL 101 is reported for comparison.

**Table 4:** EDS composition of the φ phase as a function of the temperature for samples etched for 3 min in 3 M HCl in Cyphos IL 101. The results of the sample etched for 120 min at 60°C are added for comparison.

T (°C)		O	Fe	Nd
0*	at. (%)	4.2	86.9	9
	st. dev. (%)	6.1	0.5	3.2
RT	at. (%)	4.3	86.9	8.8
	st. dev. (%)	18	0.6	2.5
40	at. (%)	4.6	86.6	8.8
	st. dev. (%)	‒	‒	‒
60	at. (%)	8.9	82.9	8.3
	st. dev. (%)	6.9	0.9	2.1
60 (120 min)	at. (%)	6.6	84.5	9.0
	st. dev. (%)	14.4	1.3	1.8

⁎ sample 0 refers to the unetched sample.

**Table 5:** EDS composition of the *n* phase as a function of the temperature for samples etched for 3 min in 3 M HCl in Cyphos IL 101. The results of the sample etched for 120 min at 60°C are added for comparison.

T (°C)		O	Nd	Dy
0*	at. (%)	23.3	61.9	14.9
	st. dev. (%)	27.5	8.4	10.7
RT	at. (%)	21.2	63.4	15.4
	st. dev. (%)	45.2	12.1	15.4
40	at. (%)	23.9	60.9	15.2
	st. dev. (%)	2.3	0.3	3.5
60	at. (%)	28.9	57.4	13.7
	st. dev. (%)	6.3	4.1	6.2
60 (120 min)	at. (%)	23.4	53.9	16.1
	st. dev. (%)	59.6	8.2	10

⁎ sample 0 refers to the unetched sample.

**Fig. 6:**
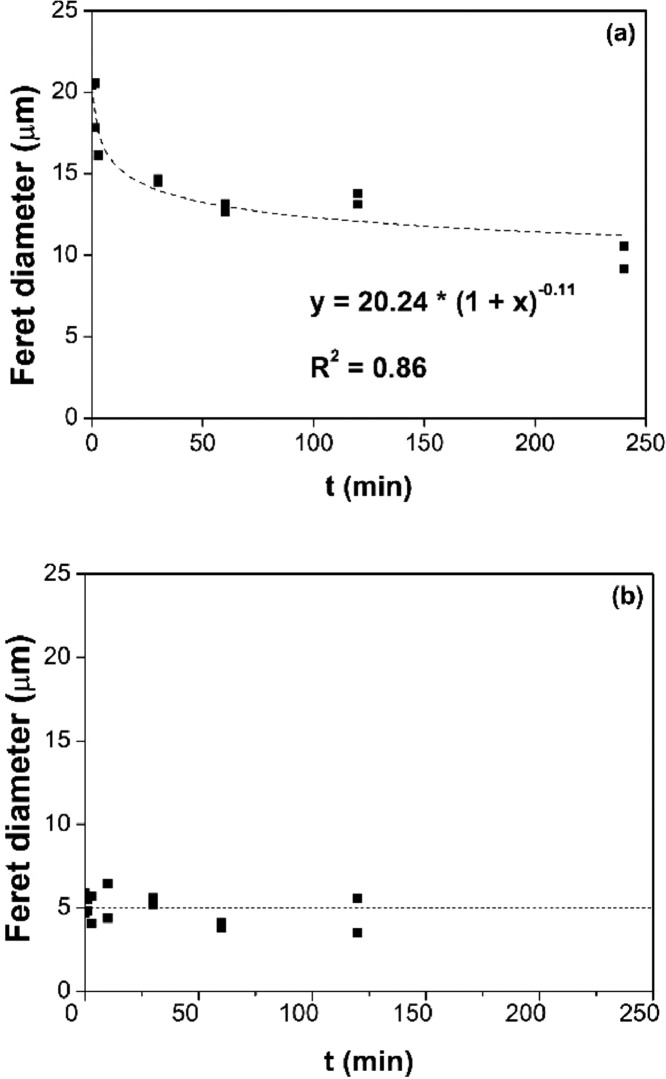
Feret diameter (μm) as a function of time, t (min) of Nd‒Fe‒B magnets etched at room temperature with 3 M HCl in Cyphos IL 101, for (a) the φ phase; and (b) for the *n* phase.

**Fig. 7:**
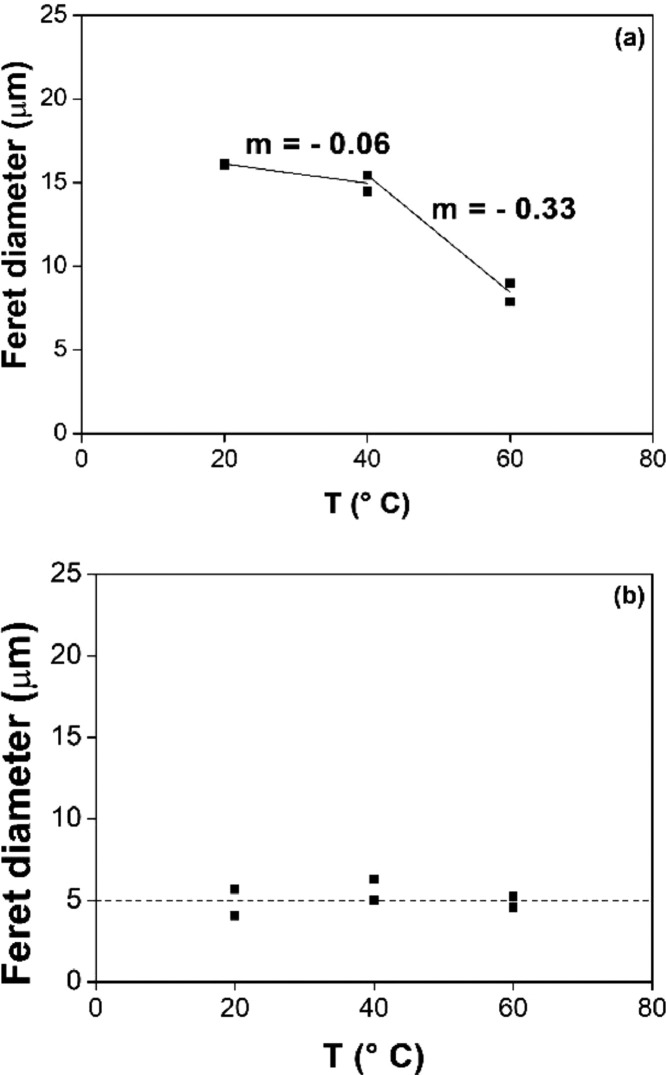
Feret diameter, (μm), as a function of the temperature, T (°C) of Nd‒Fe‒B magnets etched for 3 min with 3 M HCl in Cyphos IL 101, for (a) the φ phase; and (b) for the *n* phase.

**Fig. 8:**
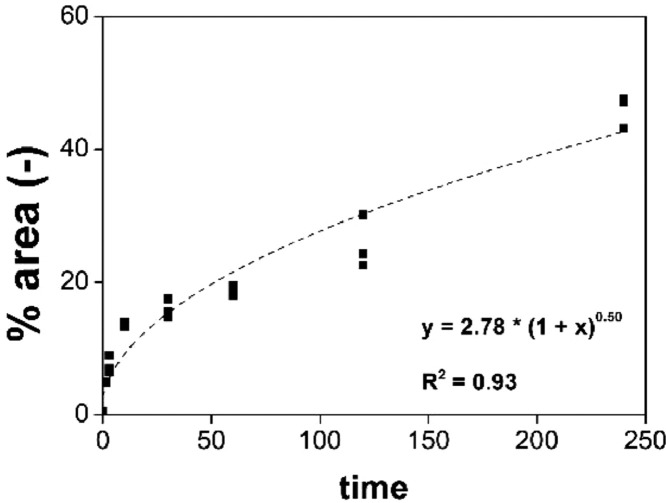
Etched area, % area (-), as a function of time, t (min) of Nd‒Fe‒B magnets etched at room temperature with 3 M HCl in Cyphos IL 101

**Fig. 9:**
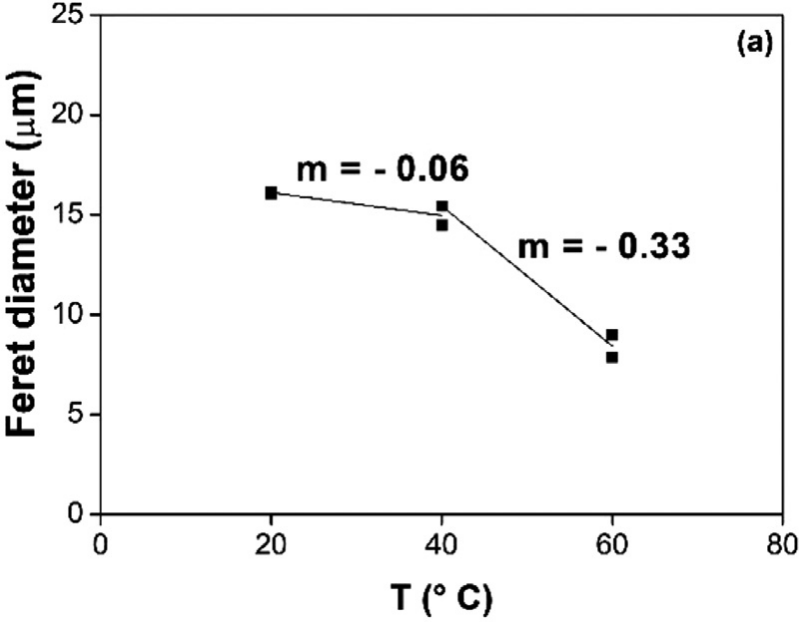
Etched area, % area (-), as a function of temperature, T (°C) of Nd‒Fe‒B magnets etched for 3 min with 3 M HCl in Cyphos IL 101.

Fig. 6, Fig. 7, Fig. 8, Fig. 9 report the fitting of the data obtained by image analysis with the software ImageJ. In particular, Fig. 6 (a) and (b) show the Feret diameter (μm) – of (a) the φ phase and of (b) the n phase – as a function of the etching time of Nd‒Fe‒B magnets, etched at room temperature with 3 M HCl in Cyphos IL 101. Fig 7 (a) and (b) depict the Feret diameter (μm) – of (a) the φ phase and of (b) the n phase – as a function of temperature of Nd‒Fe‒B magnets etched for 3 min with 3 M HCl in Cyphos IL 101. Fig. 6 (b) and 7 (b) report a constant Feret diameter, at any experimental condition, for the particles of the n phase. Fig. 8 depicts the etched area, %area (-), as a function of the etching time of Nd‒Fe‒B magnets etched at room temperature with 3 M HCl in Cyphos IL 101; whereas Fig. 9 shows the etched area, %area (-), as a function of the temperature of Nd‒Fe‒B magnets etched for 3 min with 3 M HCl in Cyphos IL 101.

## 2 Experimental Design, Materials, and Methods

• Chemicals

The ionic liquid trihexyl(tetradecyl)phosphonium chloride (Cyphos® IL 101, [C101][Cl]) was purchased from Cytec (Brussels, Belgium). Concentrated HCl (37 wt.%) was purchased from VWR (Haasrode, Belgium), while D_2_O (99.99%) from Sigma-Aldrich (Diegem, Belgium). Technical grade ethanol, propanol and acetone were purchased from Merck (Darmstadt, Germany). Nd‒Fe‒B magnets were kindly provided by Magneti Ljubljana. They were never magnetized because rejected after quality control, but would correspond to the Remag 31 A with nominal values of: remanence, *B_r_* = 1.16 T; coercivity, *H_cB_* = 880 kA m^−1^ and energy product, (BH)_max_ = 250 kJ m^−3^. These magnets are produced according to the standard process for Nd‒Fe‒B magnets [2], with the exception that isostatic pressing is not applied and that the sintering is carried out together with a heating treatment.

• Etching tests

Cyphos IL 101 saturated with HCl served as the etching agent. The solution was prepared by contacting 50 mL of HCl (37%) with 5 g of Cyphos IL 101. This mixture was biphasic and part of the HCl was extracted to the ionic liquid (IL) with a final concentration of 3.0 M acid in the IL phase. This phase was separated and used as etching agent. The effect of the acid concentration was studied by also testing Cyphos IL 101 containing 1.2 M HCl. In the latter case, the etching agent was prepared by volumetrically diluting 10 times 37 wt. % HCl in the IL. In this way, a homogeneous mixture is obtained, which is applied as etching agent. Besides the effects of the concentration of the acid, the effect of the time and of the temperature on the etching mechanism were also investigated.

Before etching, the magnets were polished using standard metallographic methods to expose the microstructure to the etching agent and to the microscope. The surface was first scratched with a Struers Rotopol-15 machine using Struers waterproof SiC polishing papers with grit (in order of use): P#220, P#600, P#1000, P#2400 and P#4000. A step of finer polishing was then performed, using a Struers Labopol-5 and a diamond DP-paste P of grain size (in order of use) 3, 1 and ¼ μm. The conditions applied with both devices were: water-off polishing, ω = 150 rpm and propanol as lubricant. Whole magnets were polished without any further treatment, since they are big enough to be easily handled. To minimize the oxidation of the surface by air in between the different steps, the samples were kept under vacuum.

For the etching tests, the polished surface of a magnet was put into contact with a droplet of the etching agent, Cyphos IL 101 saturated with HCl, in a Petri dish for a fixed time (from 1.5 min to 4 h, at room temperature) and then washed with acetone. For the temperature tests, the Petri dish was heated to 40 or 60°C for 3 min, controlled by a thermometer. The samples were analyzed via electron microscopy and image analysis. Scanning electron microscope (SEM) pictures and energy-dispersed spectra (EDS) were collected with a JEOL JSM 5800 microscope, operating at 20 kV. The polished samples were made conductive by spraying a carbon layer on them using a Balzer SCD 050 sputter coater. The EDS analysis were collected as average on at least 3 points per each SEM picture.

• Image analysis

**Fig. 10:**
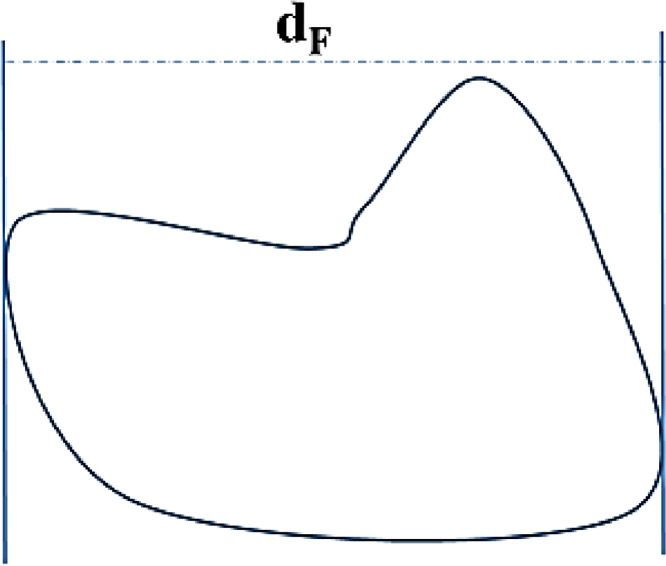
Feret diameter, d_F_, of a random particle.

Image analysis supported the mere observation of SEM pictures and the EDS analysis to understand the reactivity of the three phases η, *n* and φ of the Nd‒Fe‒B magnets. Interestingly, the literature never considered image analysis to validate or discuss the data about corrosion of Nd‒Fe‒B magnets obtained from electron microscopy. A commercial software, ImageJ®, was used for the image analysis [3]. Two data were analysed: the Feret diameter and the percentage of etched area, %area. The Feret diameter is defined as the distance between the two parallel planes that delimit the object and are perpendicular to a specific direction, with respect to which the measure is given. It is determined using the projections of a three-dimensional object on a two-dimensional plane. A graphical explanation of the Feret diameter is reported in Fig. 10. The SEM images were converted to 8-bit grayscale, from 0 to 255 number of grey ranges. Simple linear scaling was applied. A threshold was set to define the area to analyse: when the Feret diameter was measured, the threshold referred to the particles, whereas when the %area was measured the threshold referred to the void spaces. In the latter case, dimples that might have been originated by the polishing rather than by the etching were unavoidably considered as well. The error was accounted to be mostly ≤5%. However, for measurements uniformity, the dimples area was excluded using the “Set Measurements” options. Three SEM pictures for each set of temperature or time conditions were examined to minimize errors due to the quality of the pictures or to the sensitivity of the operator. Moreover, two operators carried out the image analysis and their results were averaged.

The scale set for the measurement was based on the scale of the SEM analysis, paying attention on the fact that the images were in 1:1 aspect ratio. Area fraction images in threshold mode is the fraction, in percentage, of pixels highlighted by the threshold command. The fitting of the curves Feret diameter or %area vs time or temperature was done using the Origin® software. The curves were split in two fragments, based also on the SEM images, to better fit them with a super-linear, power law or a linear law.

## Declaration of Competing Interest

The authors declare that they have no known competing financial interests or personal relationships which have, or could be perceived to have, influenced the work reported in this article.

## Appendix Supplementary materials

Image, application 1
